# Numerical characterization of regenerative axons growing along a spherical multifunctional scaffold after spinal cord injury

**DOI:** 10.1371/journal.pone.0205961

**Published:** 2018-10-26

**Authors:** Weiping Zhu, Han Zhang, Xuning Chen, Kan Jin, Le Ning

**Affiliations:** Shanghai Institute of Applied Mathematics and Mechanics, Shanghai University, Shanghai, People's Republic of China; University of Toronto, CANADA

## Abstract

Spinal cord injury (SCI) followed by extensive cell loss, inflammation, and scarring, often permanently damages neurological function. Biomaterial scaffolds are promising but currently have limited applicability in SCI because after entering the scaffold, regenerating axons tend to become trapped and rarelyre-enter the host tissue, the reasons for which remain to be completely explored. Here, we propose a mathematical model and computer simulation for characterizing regenerative axons growing along a scaffold following SCI, and how their growth may be guided. The model assumed a solid, spherical, multifunctional, biomaterial scaffold, that would bridge the rostral and caudal stumps of a completely transected spinal cord in a rat model and would guide the rostral regenerative axons toward the caudal tissue. Other assumptions include the whole scaffold being coated with extracellular matrix components, and the caudal area being additionally seeded with chemoattractants. The chemical factors on and around the scaffold were formulated to several coupled variables, and the parameter values were derived fromexisting experimental data. Special attention was given to the effects of coating strength, seeding location, and seeding density, as well as the ramp slope of the scaffold, on axonal regeneration. In numerical simulations, a slimmer scaffold provided a small slope at the entry “on-ramp” area that improved the success rate of axonal regeneration. If success rates are high, an increased number of regenerative axons traverse through the narrow channels, causing congestion and lowering the growth rate. An increase in the number of severed axons (300–12000) did not significantly affect the growth rate, but it reduced the success rate of axonal regeneration. However, an increase in the seeding densities of the complexes on the whole scaffold, and that in the seeding densities of the chemoattractants on the caudal area, improved both the success and growth rates. However, an increase in the density of thecomplexes on the whole scaffold risks an over-eutrophic surface that harms axonal regeneration.Although theoretical predictions are yet to be validated directly by experiments, this theoretical tool can advance the treatment of SCI, and is also applicable to scaffolds with other architectures.

## Introduction

Spinal cord injury (SCI) typically results in a permanent loss of neurological function below the level of the injury [1,2]. Recovery from SCI is poor owing to weak intrinsic growth capacity of axons and an inhibitory microenvironment, as well as a lack of suitable growth substrates and growth-stimulating factors, that limit axonal regeneration. Many efforts have been made to overcome these impediments [3,4,5,6,7], including implanting of cells [8,9] and/or biomaterials [10,11,12,13,14,15,16,17], delivery of growth factors and degradation of inhibitory matrix molecules [18,19,20,21,22,23,24,25], activation of an intrinsic growth program [26], and stabilization of growth cones and the axonal cytoskeleton [27]. Biomaterial scaffolds that can fill or bridge a lesion cavity, provide a substrate for cell seeding, offer physical guidance to regenerating axons or act as a vehicle for drug delivery are highly promising for cellular and molecular regenerative therapies [19]. However, the use of biomaterial scaffolds as a bridge for SCI repair is complicated; for instance, a popular biomaterial scaffold has tunnels/linear pores, which could guide regenerating axons along these tunnels [14,19]. However, significant congestion was observed at the entry points to the scaffold, which lowered the number of axons entering the pores [19]. If pores were coated or seeded with extracellular components (ECM) components [14] or cells [19], the axons were likely to enter the tunnels from both rostral and caudal ends, but became trappedwithin them [14], unless an additional injection of cells and/or growth factors close to either side of the bridge was administered [18,19], after which some of the trapped axons would be attracted to the injection site and re-enter host tissues [19]. These issues involve the architecture of the “on-ramp” and “off-ramp” parts of the bridge[3], and the concentration gradients of the molecules released from the cells around and seeded on the scaffold, and their influence on successful growth rates of regenerative axons have not yet been clarified.As an analytical tool, mathematics can be used to address many aspects of these issues, for example, the role of slopes at the on-ramp part of a scaffold. When a scaffold with linear pores is used, the axons whose growth cones face to the pores can easily enter the pores owing to the zero entry slope for them, whereas for axons beside the pores, an abrupt or infinite (mathematically) slope exists, which obstructs the axons and leads to congestion. Therefore, theoretical models which address the physical basis underlying the regulatory effect of ligand gradients on axonal growth cone motility are highly desirable, astheyhave been previously used for studying axonal growth during neural development [28,29,30,31]. Axonal growth or regrowth follow the same principle, both during development and following injury. A difference between them, however, has been observed in the level of factors present in their microenvironments [1,2]. Therefore, a theoretical model for axonal growth during development should be modified for studying SCI.Previously, in an initial study [32], we numerically demonstrated that SCI leaves a spherical glial scar. During therapeutic treatment with Schwann cell coating, regenerating axons were shown to grow across the scar and reach their target cellsunder certain conditions.

**Fig 1:**
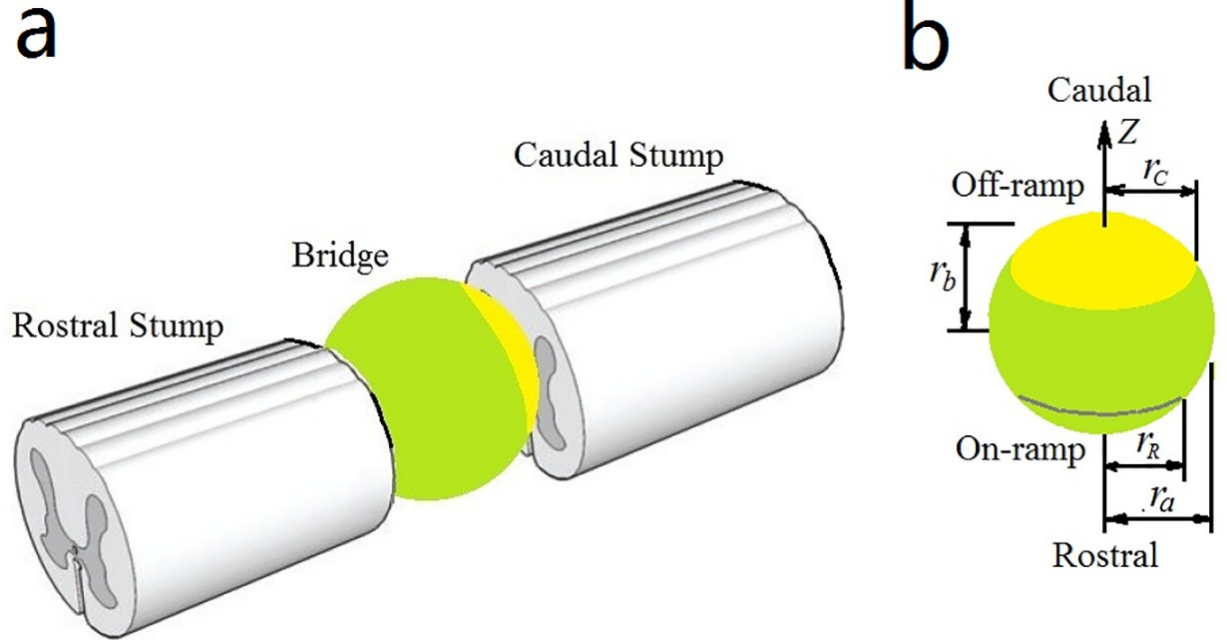
Schematics of an injured spinal cord bridged by a spherical scaffold. **a)** A complete transection of spinal cord with a gap bridged by a spherical scaffold between the rostral and caudal stumps. **b)** The scaffold architecture, coordinate system, and symbols employed in the mathematical models. The fabricated scaffold is assumed to be coated with hydroxylapatite (HA)/extracellular matrix (ECM) components (collagen I, fibronectin, and laminin I) and HA/LV-chondroitinase ABC (ChABC) over the whole surface (green). Additional HA/LV-NT-3/LV-brain-derived neurotrophic factor (BDNF) was coated on the off-ramp area (yellow).

In this study, we assumed a solid, spherical, multifunctional, biomaterial scaffold that would bridge the rostral and caudal stumps of a completely transected spinal cord in a rat model and guide the regenerative rostral axons toward the caudal tissue (Fig 1). The rostral and caudal areas of the scaffold were referred to as the entry "on-ramp" area and the exit "off-ramp" area, respectively. The whole scaffold was coated with extracellular matrix components, and the caudal area (off-ramp) was additionally seeded with chemoattractants. In this context, the chemical factors on and around the scaffold were formulated to several coupled variables, and the parameter values were derived fromexisting experimental data. The effects of axonal regeneration on the on-ramp slope of the scaffold and biomedical modifications were numerically simulatedto provide a quantitative interconnection between axonal regeneration and the biophysical properties of the components for rational design of scaffolds in tissue engineering [33], as well as contribute to a better understanding of the biological processes involved. From this model and the simulations, we can learn what constitutes a good scaffold, good entry points for regenerative axons,and the resultant concentrations of the molecules from the microenvironment and the scaffold, and how the sources of the main attractants vary along the length of the scaffold in a monotonic curve. Moreover, our model can be modified to optimize scaffolds with other architectures, includingdifferences in size and shape, in concentration of molecules inside and around the scaffold, and before and after experiments.

## Materials and methods

Materials and methods have been described primarily from the computational point of view.

### Geometry of the scaffold

The scaffold is assumed to be a rotational ellipsoid in such a coordinate system that the *z*-axis is the rotational axis and is orthogonal to the *x*- and *y*-axes (Fig 1B), and the origin *O* (0, 0, 0) is at the ellipsoid center. The lateral and longitudinal semi-axes of the ellipsoid are denoted by *r*_*a*_ and *r*_*b*_, respectively. When *r*_*a*_ and *r*_*b*_ are equal, the ellipsoid becomes a perfect sphere. Moreover, the rostral and caudal sides of the scaffold are defined as the entry on-ramp and exit off-ramp, respectively [3]. The longitudinal length of the on-ramp/off-ramp (measured from the scaffold end-point to where the rotational radius of the scaffold surface equals *r*_*R*_/*r*_*C*_) is *r*_*b*_/3. The length parameters (*r*_*a*_,*r*_*b*_, *r*_*C*_,*r*_*R*_, and others) are scaled to dimensionless values by the characteristic length *L* of the model. The parameter *r*_*a*_ is controllable, and *r*_*b*_ is fixed at 2 × *r*_*b*_× *L* = 3 mm (spanning the gap of the spinal cord). Three scaffolds were prepared: slim (*r*_*a*_×*r*_*b*_ = 0.15×0.3), round (0.3×0.3), and stocky (0.45×0.3), labeled as No. 1, No. 2, and No. 3, respectively. For brevity, all scaffolds are referred to as “spherical”, noting that when the longitudinal size *r*_*b*_ is fixed, the on-ramp/off-ramp slope is proportional to the lateral size *r*_*a*_. Correspondingly, the entry slopes of the slim, round, and stocky scaffolds are small, medium, and large, respectively. Therefore, when studying the effect of the entry slope of the scaffold on axonal regeneration, we need to vary *r*_*a*_ in the model.

#### Assumptions about fabrication, coating, and seeds of the scaffold

In the currentstudy,processing a scaffold relates to setting the model parameters. There are several existing techniques available for fabrication and modification of the scaffold, for example the poly (D,L-lactide-co-glycolide) (PLG) with a gas-foaming/particulate-leaching process [14,24]. To prepare the coating and seeds of the scaffold, hydroxylapatite (HA) nanoparticles (Sigma-Aldrich) were suspended in phosphate-buffered saline and sonicated for 1 min to dissociate the aggregates [14,24]. The nanoparticles were then complexed with several ECM components (forming HA/ECMs), including collagen I (BD Biosciences, San Jose, CA), fibronectin (Sigma-Aldrich, St. Louis, MO), and laminin I (Trevigen, Gaithersburg, MD) [22]. Lentivirus was used to encode the neurotrophic factors NT-3 (HA/LV-NT-3) and BDNF (HA/LV-BDNF) [24] and the ChABC gene (HA/LV-ChABC) [21,23,24]. The HA/ECMs and HA/virus complexes were then incubated for 10 min at 4°C and deposited onto the scaffold surface using Stripper pipette tips (Mid-Atlantic Diagnostics, Mount Laurel, NJ). The HA/ECMs and HA/LV-ChABC were coated over the entire surface, whereas HA/LV-NT-3 and HA/LV-BDNF were only deposited on theoff-ramp surface of the bridge. Although HA could firm the coating and/or seeds on the PLG material surface [24], it probably stimulates bone formation [34]; therefore, it would be best to avoid the overuse of HA. In addition, the prepared scaffold should be placed on dry ice until implantation. This scaffold could then beused to replace previous scaffolds used in a rat model for SCI repair [10,11,16], to bridgea gap of approximately 3 mm. The scaffold was considered to perform the following functions:the factors seeded on the bridge surface were localized and sustained; the on-ramp slope introduced the rostral regenerative axons onto the scaffold; the secreted ChABC cleared the growth pathways; the ECM components kept the axons on target; and the gradients of the secreted NT-3 and BDNF guided the growing rostral axons along the correct path while blocking the caudal axons.

### Scaffold surface equation and constraints for growth cones

Geometrically, the scaffold is an ellipsoid that rotates about its *z*-axis (Fig 1B). The surface equation reads as follows:

$${\left(R/r_{a}\right)}^{2}+{\left(z/r_{b}\right)}^{2}=1,\;\mathrm{with}\;R=\sqrt{x^{2}+y^{2}}.$$ (1)

Here, *x*, *y*, and *z* are the coordinates of a point on the surfaceand *R* is the distance from that point to the *z*-axis.

When an axon grows along the scaffold surface, the growth cone can be considered as a particle moving on the surface. The ECM molecules coated on the scaffold are assumed to strongly adhere to the membrane proteins of the growth cones, tethering them to the scaffold. In other words, the movements of the growth cones are constrained by Eq (1). Taking the time derivative of both sides of Eq (1), the constraint conditions of the growth cone velocity can then be obtained as follows:

$$V_{R}=-{\left(r_{a}/r_{b}\right)}^{2}V_{z}z/R,\;V_{R}=dR/dt,\;V_{z}=dz/dt,$$ (2)

$$V_{x}=dx/dt=V_{R}x/R,\;V_{y}=dy/dt=V_{R}y/R,$$ (3)

where *V*_*x*_, *V*_*y*_, and *V*_*z*_ are the velocity components of a growth cone in the *x*-, *y*-, and *z*-directions, respectively, and *V*_*R*_ is the velocity component along the rotational radius *R*. In Eq (2), *V*_*z*_ is the longitudinal velocity of the growth cone, which defines the drawing speed or growth rate of the axons. When *V*_*z*_>0, the axon is elongating. Otherwise it is retracting. *V*_*R*_ is the lateral velocity of the growth cone. When *V*_*z*_>0 and *z*<0, *V*_*R*_>0 and the growth cone progresses from the on-ramp of the scaffold. Conversely, when *V*_*z*_>0 and *z*>0, *V*_*R*_<0 and the growth cone advances to the off-ramp. In the next two subsections, we describe the drawing force and locomotive guidance of the growth cone along the scaffold.

### Equations for axonal growth

Many pieces of evidence have shown that axonal growth cones move because of chemotactic processes, biased toward (attractive chemotaxis) or away from (repulsive chemotaxis) the chemical source [35,36,37]. The chemicals that attract and repel regenerative axons are found on and around the multifunctional scaffold (this is hereafter assumed to be the case in the implanted status), and will be classified in the next subsection. The chemotactic force, which defines the attractive or repulsive action on a growth cone, is proportional to the gradient of the diffusible molecules released from the chemical source [29,32,37,38,39,40,41]. The growth cone can be regarded as a particle whose persistent velocity determines the growth rate of an axon. As axonal growth is particularly slow (approximately 0.01 μms^−1^) [35,36,37], the acceleration or inertial force can be neglected [24]. Stochastic factors can also be neglected because the chemotactic movement is highly consistent [29]. Therefore, the velocity of the growth cone is directly proportional to the chemotactic force:

$$\frac{dr_{k}^{A}}{dt}=\frac{1}{\mu }F_{k}^{A},\;k=1,2,\cdots ,N_{A},$$ (4)

$$F_{k}^{A}=\sum_{i=1}^{3}\lambda_{i}p_{i},\;p_{i}=\nabla \rho_{i}\frac{\left|\Delta r_{k}^{A}\right|}{\rho_{\sum }},\;\rho_{\sum }=\sum_{i=1}^{3}\rho_{i},\;i=1,2,3,$$ (5)

where *k* and *N*_*A*_ are the number and total number of regenerative axons/growth cones, respectively. $r_{k}^{A}$ ($=x_{k}^{A}i+y_{k}^{A}j+z_{k}^{A}k$, where **i**, **j**, and **k**are the unit vectors in the Cartesian coordinate system) is the position of the *k*-th growth cone at time *t*. μ is the dynamic viscosity coefficient. $F_{k}^{A}$ ($=F_{xk}^{A}i+F_{yk}^{A}j+F_{zk}^{A}k$) is the result of the chemotactic forces from all types of chemotactic-related molecules (CRMs) acting on the *k*-th growth cone at *t*. The CRMs will be classified into three types in the next subsection, where *i* is the number of types of CRMs, and subscript *i*denotes the *i*-th type of CRMs (CRMs-*i*). The chemotactic force [Eq (5)] is defined as a dimensionless vector (**p**_*i*_) with a proportionality constant (*λ*_*i*_). In this equation, *ρ*_*i*_ is the concentration of CRMs-*i* at $r_{k}^{A}$ and *t*; ∇*ρ*_*i*_ is the gradient of *ρ*_*i*_ (where ∇ = **i**∂/∂*x*+**j**∂/∂*y*+**k**∂/∂*z* is the Hamiltonian operator); $\left|\Delta r_{k}^{A}\right|=\sqrt{\Delta x^{2}+\Delta y^{2}+\Delta y^{2}}$ is the scalar difference of $r_{k}^{A}$ across the width of the *k*-th growth cone; and *ρ*_∑_ is the sum of *ρ*_*i*_ over all types of CRMs. Note that the scalar of **p**_*i*_ [Eq (5)] simplifies to Δ*ρ*_*i*_/*ρ*_∑_, which expresses the relative difference of *ρ*_*i*_, with Δ*ρ*_*i*_ being the absolute difference of *ρ*_*i*_ across the distance $\left|\Delta r_{k}^{A}\right|$. In practice, the average width of the growth cone can be considered as $\left|\Delta r_{k}^{A}\right|$ ~ 10μm [29,38]. Therefore, the gradient and relative difference of the chemotactic concentration are mathematically linked through **p**_*i*_. The corresponding proportionality constant *λ*_*i*_ then acquires a clear physical meaning of force per unit length. This model considerably reduces the growth-rate distortion of the growth cone when close to the target cells. Note that in the one-dimensional single-component problem [29,38,39], **p**_*i*_ reduces to *p* = Δ*ρ*/*ρ*, which defines the concentration gradient in some biophysical areas.

Finally, from Eqs (2), (4) and (5), the drawing speed *V*_*z*_ of the *k*-th axon can be expressed as follows:

$$V_{zk}=\frac{dz_{k}^{A}}{dt}=\frac{1}{\mu }F_{zk}^{A},k=1,2,\ldots ,N_{A},$$ (6)

$$F_{zk}^{A}=\sum_{i=1}^{3}\lambda_{i}\frac{\partial \rho_{i}}{\partial z}\cdot \frac{\Delta z}{\rho_{\sum }},\;\rho_{\sum }=\sum_{i=1}^{3}\rho_{i},$$ (7)

where Δ*z* is the cone’s width along the *z*-axis. The other symbols have been defined in Eqs (4) and (5).

### Evolution equations for the chemotactic-related molecules

Based on the chemical coarse-graining concept, the chemotactic-related molecules (CRMs) on and around the multifunctional scaffold can be classified into three types. The first group (denoted as Type 1 or CRMs-1) comprises the chemoattractants for axonal growth, such as NT-3 and BDNF [35,36], secreted by seeded HA/LV-NT-3 and HA/LV-BDNF at the off-ramp area (**Fig 1B**; yellow). The second group (Type 2 or CRMs-2) comprises the chemorepellents Nogo-60, myelin-associated glycoprotein (MAG), and oligodendrocyte-myelin glycoprotein (OMG) released or upregulated in response to the injured tissues [42,43,44,45]; and the remnant chondroitin sulfate proteoglycans (CSPGs) that are not neutralized by ChABC [1,2,9], secreted by seeded HA/LV-ChABC over the entire scaffold surface. Finally, the third group (Type 3 or CRMs-3) includes molecules released from coated HA/ECM components (collagen I, fibronectin, and laminin I) on the scaffold, which support axonal growth [24,35,36]. The mixed concentrations of CRMs-1, CRMs-2, and CRMs-3 are denoted by *ρ*_1_, *ρ*_2_, and *ρ*_3_, respectively. Among these groups, CRMs-1 plays the leading role in promoting axonal regeneration, CRMs-2 inhibits axonal growth, and CRMs-3 plays a supplementary role in stabilizing axonal growth. In addition, CRMs-1 and CRMs-2/3 might crosstalk via signal transduction [46]. The diffusions and reactions of *ρ*_1_, *ρ*_2_, and *ρ*_3_ obey Fick’s first law [28,29]. Considering these CRM mechanisms in SCI regeneration, the evolution equations for *ρ*_1_, *ρ*_2_, and *ρ*_3_ are given as follows:

$$\frac{\partial \rho_{1}}{\partial t}=D_{1}\nabla^{2}\rho_{1}-k_{-1}\rho_{1}+\sum_{j=1}^{N_{T}}\sigma_{1}\delta (r-r_{j}^{T}),$$ (8)

$$\frac{\partial \rho_{2}}{\partial t}=D_{2}\nabla^{2}\rho_{2}-k_{-2}\rho_{2}+\sum_{k=1}^{N_{A}}\sigma_{2}(\rho_{1})\delta (r-r_{k}^{A}),$$ (9)

$$\frac{\partial \rho_{3}}{\partial t}=D_{3}\nabla^{2}\rho_{3}-k_{-3}\rho_{3}+\sum_{k=1}^{N_{A}}\sigma_{3}(\rho_{1})\delta (r-r_{k}^{A}).$$ (10)

In Eqs (8)–(10), *ρ*_*i*_(*i* = 1,2,3) are functions of both time (*t*≥0) and space (on and around themultifunctional scaffold), each point is represented by a position vector, **r** = *x***i**+*y***j**+*z***k**, where *x*, *y*, and *z* are the coordinates and **i**, **j**, and **k** are their corresponding unit vectors in the Cartesian coordinate system. ∇^2^ = ∂^2^/∂*x*^2^+∂^2^/∂*y*^2^+∂^2^/∂*z*^2^ is the Laplace operator. *D*_*i*_ and *k*_−*i*_ are the diffusion and attenuation coefficients of *ρ*_*i*_, respectively. ∑ denotes the summation of all point-source terms. *δ*(⋅) is the Dirac delta function, defined as *δ*(0) = 1 and *δ*(else) = 0. *N*_*A*_ is the total number of severed axons before their repair. *N*_*T*_ is the total number of CRMs-1 point sources, whichdepends on the density of HA/LV-NT-3 and HA/LV-BDNF seeded in the off-ramp area (**Fig 1B**; yellow) and on the chemical coarse-graining model. $r_{j}^{T}$ (immobile) is the position of the *j*-thpoint source of CRMs-1 (where *j* = 1, 2, …, *N*_T_), and $r_{k}^{A}$ is the time-dependent position of the *k*-th growth cone capped on the regenerating axons (*k* = 1, 2, …, *N*_A_). *σ*_1_, *σ*_2_(*ρ*_1_), and *σ*_3_(*ρ*_1_) are the point-source release rates of CRMs-1, CRMs-2, and CRMs-3, respectively. Note that *σ*_2_(*ρ*_1_) and *σ*_3_(*ρ*_1_) are the functions of *ρ*_1_, which indicates crosstalk or interactions betweenCRMs-1, CRMs-2, or CRMs-3. We can reduce the functions to *σ*_2_ = *σ*_20_(1−*R*_*L*_) and *σ*_3_ = *σ*_30_*R*_*L*_ with *R*_*L*_ = *ρ*_1_/(*K*_d_+*ρ*_1_), where *σ*_20_ and *σ*_30_ are the normal release rates of CRMs-2 and CRMs-3 point sources, respectively, and *K*_d_ is the dissociation constant [32,38]. *R*_*L*_ defines receptor-ligand associativity on the growth cone membrane. *σ*_20_(1−*R*_*L*_) and *σ*_30_*R*_*L*_ reflect the competitive relationship between CRMs-2 and CRMs-3. Eqs (8)–(10) are multi-component dynamic reaction-diffusion equations with nonlinear coupling of the point sources.

### Numerical methods

**Fig 2:**
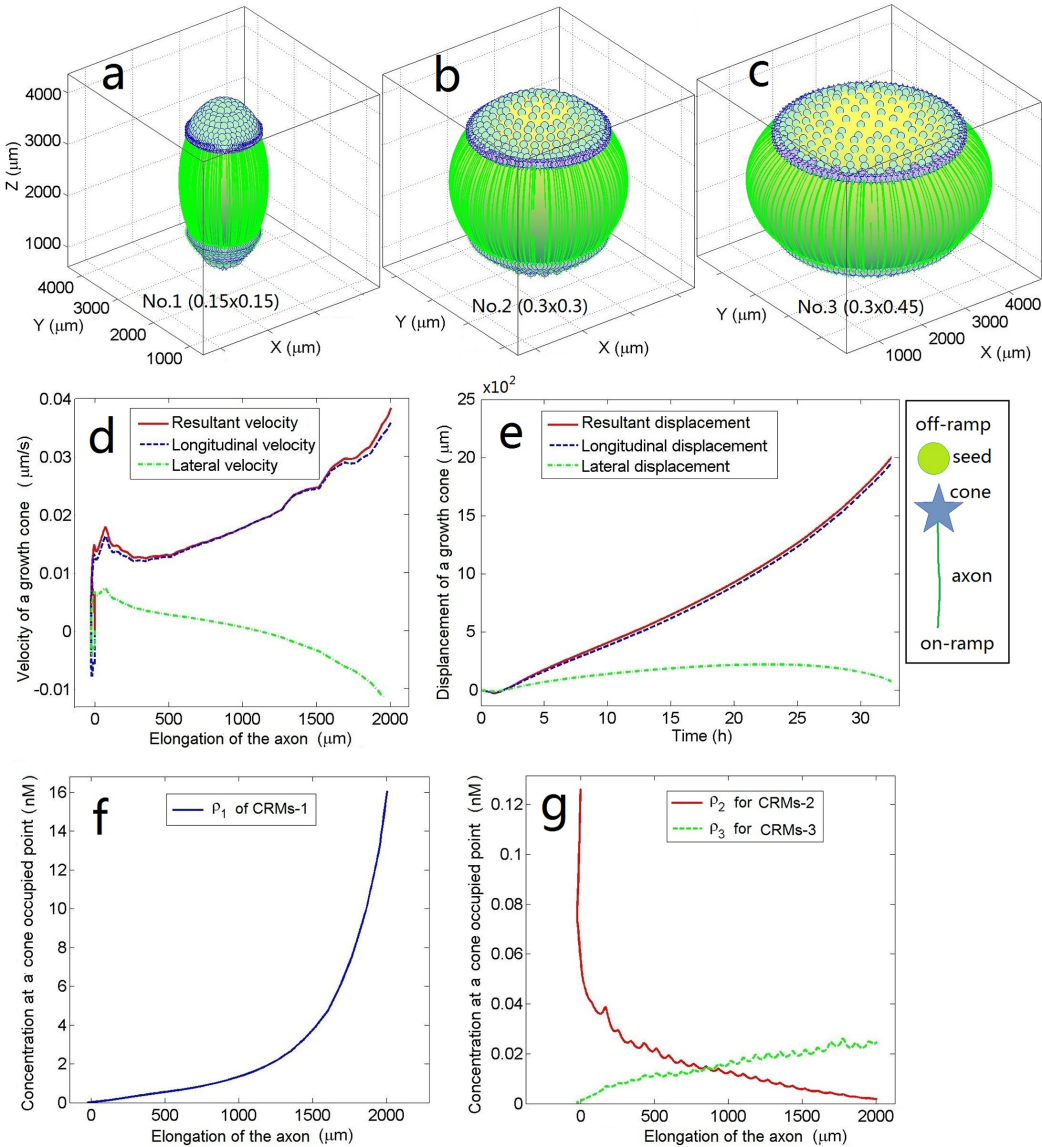
Three test scaffolds of different lateral sizes and some products. **a)–c)**The scaffolds and attached regenerated axons are separated and extracted from the models after calculation. In scaffolds No. 1, No. 2, and No. 3, the lateral semi-axis is *r*_*a*_ = 0.15, 0.3, and 0.45 (or 0.75, 1.50, and 2.25 mm), respectively. The longitudinal semi-axis is *r*_*b*_ = 0.3(or 1.5 mm) in all cases, and the number of severed axons is *N*_*A*_ = 300 in each calculation. The green bubbles with number *N*_*T*_ = 300 and density *γ*_1_ = 0.1 μm^−2^ embedded in the upper part (or the caudal/off-ramp) of each scaffold represent the coarsening point sources (or target cells) for CRMs-1 (two other seed types with densities *γ*_2_ = *γ*_3_ = 0.1 μm^−2^ also cover the whole surface of each scaffold but are not shown). The green longitudinal lines (slightly fascicled) stemming from the lower part (the rostral/on-ramp) of each scaffold represent the regenerated axons (numbering 0.78 *N*_*A*_, 0.7 *N*_*A*_, and 0.58 *N*_*A*_ in **a**, **b**, and **c**, respectively, corresponding to axonal regeneration success rates of 0.78, 0.7, and 0.58, respectively). **d)–g)** Results related to a typical regenerating axon taken from the calculation of scaffold No. 1 (qualitatively similar results are obtained for the other two scaffolds). At the beginning, when the axon is at the on-ramp, its growth is unstable and some retraction is observed (**d** and **e**) because the concentrations of the positive factors CRMs-1 (**f**) and CRMs-3 are low (**g**; green), while those of the inhibitoryfactors are high (**g**; red). This situation is reversed as the axon approached the off-ramp.

Eqs (1)–(10) comprise the mathematical model of regenerative axons growing along the spherical multifunctional scaffold where their status is as shown in Fig 1A or Fig 2A–2C. The source terms in Eqs (9) and (10) are nonlinearly coupled with Eq (8) and Eqs (4)–(7) through point $r_{k}^{A}$ that tracks the growth cone. Therefore, Eqs (4)–(10) comprise a set of coupled nonlinear differential equations that can only be solved numerically.

The model is solved using three methods in three steps. The first step solves Eqs (8)–(10) for *ρ*_1_, *ρ*_2_, and *ρ*_3_ using the lattice Boltzmann method (LBM) [47,48,49,50]. Using a central difference and interpolation scheme, the second step solves the gradients of *ρ*_1_, *ρ*_2_, and *ρ*_3_ at the growth cone, and the axonal growth rates, using Eqs (6) and (7) and Eqs (2) and (3). Finally, the axonal growth path is obtained by integrating Eqs (6) and (7) and Eqs (2) and (3) using Euler’s method.

In the numerical simulation, we changed the lateral size of the scaffold (i.e., we changed the slope of the on-ramp/off-ramp) and the seeding densities of the HA/ECM components HA/LV-ChABC, HA/LV-NT-3, and HA/LV-BDNF. The simulation recorded the growth rates of the regenerating axons, and tested whether the regenerating axons could grow across the scaffold and finally connect with their targets.

### Consideration of the parameter values

Based on data from in vitro experiments [35,36,37], we can estimate the order of magnitude of each parameter. The estimates are as follows: growth cone width $\left|\Delta r_{k}^{A}\right|$ is approximately 10–20μm; axonal growth rate is approximately 0.01μms^-1^;and the diffusion coefficient *D*_1_ and dissociation constant *K*_d_ of CRMs-1 (e.g. NGFs) are approximately 10–50μm^2^s^-1^and 1nM, respectively. In addition, the range of concentrations for NGFs is ~(0.01–10)*K*_d_, and the minimum relative concentration difference to which the growth cone can respond [35,36,37,38,39], |Δ*ρ*_*i*_/*ρ*_*i*_|, is ~1%. However, the values of many parameters cannot be recognized, including the point-source release rate *σ*_*i*_, the attenuation coefficients *k*_−*i*_, the force proportionality constant *λ*_*i*_, and the viscosity coefficient *μ*. Given that the diffusion velocity $k_{-1}\sqrt{D_{1}/k_{-1}}$ of CRMs-1 should exceed the constant velocity of the growth cone, the absolute concentration should satisfy *ρ*_1_≥*K*_d_ and the relative concentration difference should satisfy $|\Delta \rho_{1}/\rho_{1}|=\left|\Delta r_{k}^{A}\right|/\sqrt{D_{1}/k_{-1}}\geq 1\%$ at the diffusion radius $\sqrt{D_{1}/k_{-1}}$. Under these conditions, the attenuation coefficient was estimated as *k*_−1_≥2.0×10^−6^. At the point source, we should have *ρ*_1_ = *eK*_d_+*σ*_1_/*k*_−1_≤10 *K*_d_ so that we can estimate the point-source release rate as *σ*_1_ = 1.82×10^−5^−3.64×10^−4^ nMs^-1^. From $\rho_{1}=K_{d}\exp(1-r_{\max}/\sqrt{D_{1}/k_{1}})\geq 0.01K_{d}$, the most effective diffusion radius was then estimated as *r*_max_ = 5.6 $\sqrt{D_{1}/k_{-1}}$. Finally, in the concentration field based on the above data, the axonal growth rate was assumed to be 0.0025–0.05μms^-1^. After many numerical trials, the number of *λ*_1_/*μ* was approximated as 1. The parameter values of CRMs-2 and CRMs-3 were set based on those of CRMs-1. All parameter values are listed in Table 1.

**Table 1:** Parameters used in simulations.

Symbols	Definitions	Values	From
*A*_cone_	ventral area of a growth cone	100 μm^2^	[36,37,38,39]
*A*_C_	area of the caudal region of the scaffold	related to scaffold size	Fig 1
*D*_1_	diffusion coefficient of CRMs-1	50 μm^2^s^−1^	[38], Eq (8)
*D*_2_	diffusion coefficient of CRMs-2	10 μm^2^s^−1^	Eq (9)
*D*_3_	diffusion coefficient of CRMs-3	10 μm^2^s^−1^	Eq (10)
*k*_−1_	attenuation coefficient of CRMs-1	1.25×10^−3^s^-1^	Eq (8)
*k*_−2_	attenuation coefficient of CRMs-2	2.5×10^−3^s^-1^	Eq (9)
*k*_−3_	attenuation coefficient of CRMs-3	2.5×10^−3^s^-1^	Eq (10)
*K*_d_	dissociation constant of CRMs-1/2/3	1 nM	[38], Eqs (9) and (10)
*L*	side length of the computation domain	5000 μm	Fig 1
*N*_A_	total/equivalent number of severed axons	300–12000	Eqs (9) and (10)
*N*_T_	total/equivalent number of target cells	300–12000	Eq (8)
*r*_*a*_	semi-axes of ellipsoid/scaffold	0.15–0.45, scaled by *L*	Fig 1B
*r*_*b*_	semi-axes of ellipsoid/scaffold	0.3, scaled by *L*	Fig 1B
*r*_*C*_	maximum rotational radius of scaffold caudal	0.75*r*_*a*_	Fig 1B
*r*_*R*_	maximum rotational radius of scaffold rostral	0.75*r*_*a*_	Fig 1B
$\left|\Delta r_{k}^{A}\right|$	width of a growth cone	10 μm	[38], Eq (5)
$\gamma_{1}^{0}$	normal density of the point source of CRMs-1	0.1 μm^-2^	Eq (8)
$\gamma_{2}^{0}$	normal density of the point source of CRMs-2	0.1 μm^-2^	Eq (9)
$\gamma_{3}^{0}$	normal density of the point source of CRMs-3	0.1 μm^-2^	Eq (10)
*λ*_1_	force proportionality constant of CRMs-1	1 nN μm^-1^	Eq (5)
*λ*_2_	force proportionality constant of CRMs-2	1 nN μm^-1^	Eq (5)
*λ*_3_	force proportionality constant of CRMs-3	1 nN μm^-1^	Eq (5)
*μ*	dynamic viscosity coefficient	1 nN μm^-2^s	Eq (4)
*σ*_1_	normal release rate of a CRMs-1 point source	6.75×10^−5^nMs^-1^	Eq (8)
*σ*_20_	normal release rate of a CRMs-2 point source	6.75×10^−5^nMs^-1^	Eq (9)
*σ*_30_	normal release rate of a CRMs-3 point source	6.75×10^−5^nMs^-1^	Eq (10)
*σ*_eq1_	equivalent *σ*_1_	*γ*_1_*A*_C_*σ*_1_/*N*_A_	Eq (8)
*σ*_eq2_	equivalent *σ*_2_	*γ*_2_*A*_cone_*σ*_2_	Eq (9)
*σ*_eq3_	equivalent *σ*_3_	*γ*_3_*A*_cone_*σ*_3_	Eq (10)
*η*_1_	density scale factor for CRMs-1	$\eta_{1}=\gamma_{1}/\gamma_{1}^{0}$, controllable	Eq (8)
*η*_2_	density scale factor for CRMs-2	$\eta_{2}=\gamma_{2}/\gamma_{2}^{0}$, controllable	Eq (9)
*η*_3_	density scale factor for CRMs-3	$\eta_{3}=\gamma_{3}/\gamma_{3}^{0}$, controllable	Eq (10)

### Equivalent number of seeds on the scaffold and the point-source release rate

To save computational resources, we employed coarse-grained processing, i.e., making up an equivalent number of seeds (point sources) for calculation that was smaller than that of seeding on the scaffold during fabrication. Because the fabrication process controls the densities of HA/ECMs/LV-ChABC, HA/LV-NT-3, and HA/LV-BDNF seeded on the scaffold, the densities of the point sources of CRMs-1, CRMs-2, and CRMs-3 can be quantified as $\gamma_{1}=\eta_{1}\gamma_{1}^{0}$, $\gamma_{2}=\eta_{2}\gamma_{2}^{0}$, and $\gamma_{3}=\eta_{1}\gamma_{3}^{0}$, respectively, where $\gamma_{1}^{0}$, $\gamma_{2}^{0}$, and $\gamma_{3}^{0}$ are the normal point-source densities of CRMs-1, CRMs-2, and CRMs-3 (in μms^-2^) respectively, and *η*_1_, *η*_2_, and *η*_3_ are their respective controllable scale factors. The areas of the scaffold off-ramp (caudal side) and the ventral portion of a growth cone are denoted by *A*_C_ and *A*_cone_, respectively. Thus, the total number of CRMs-1 point sources in the off-ramp area is *γ*_1_*A*_C_, i.e., *N*_T_ = *γ*_1_*A*_C_ in Eq (8). Meanwhile, the total numbers of CRMs-2 and CRMs-3 connected to a single growth cone are *γ*_2_*A*_cone_ and *γ*_3_*A*_cone_, respectively. In Eqs (9) and (10), *N*_A_is the total number of axons severed in an injury event. In a complete transection rat model, *N*_A_ might exceed 10^4^ (estimated from the cross-sectional area ratio of an axon to the spinal cord). Because the exact value *N*_A_ was unavailable, we ranged *N*_A_ from 300 to 12000 in the simulations. Correspondingly, *N*_T_ and *σ*_1_ in Eq (8) were replaced by their equivalent values *N*_eqT_ and *σ*_eq1_, respectively. Considering *N*_eqT_ = *N*_A_ and applying the mass conservation principle, we obtained *σ*_eq1_ = *γ*_1_*A*_C_*σ*_1_/*N*_A_, where *A*_C_/*N*_A_ = *A*_C_/*N*_eqT_ represents the area occupied by each equivalent point source. Here, the point sources were assumed to be evenly distributed on the off-ramp of the scaffold. Similarly, *σ*_2_ and *σ*_3_in Eqs (9) and (10) were replaced by their equivalent values *σ*_eq2_ = *γ*_2_*A*_cone_*σ*_2_ and *σ*_eq3_ = *γ*_3_*A*_cone_*σ*_3_, respectively, where *A*_cone_ represents the area on the scaffold surface covered by one moving growth cone at that moment and *γ*_2_*A*_cone_ and *γ*_3_*A*_cone_ represent the original numbers of CRMs-2 and CRMs-3 point sources on one *A*_cone_, respectively. After an equivalent transformation, each *A*_cone_ includes two point sources: a CRMs-2 point source with release rate *σ*_eq2_ and a CRMs-3 point source with release rate *σ*_eq3_. Both releases activate at the location of the cone and deactivate when the cone departs. This activation-deactivation process of the point sources is modeled by the Dirac delta function *δ*(⋅) in Eqs (8)–(10).

In the simulations, we confined the architecture and mathematics of the model to a cubical compartment with side length *L* = 5000 μm and imposed absolute boundary conditions. The D3Q15 mode [32,47] with a 64 ×64 ×64 lattice was applied to LBM simulations. MATLAB 7.11.0 (The MathWorks, Inc.) was employed to programthesimulations and run them on ThinkServer TD350 (Lenovo Group Ltd). All parameters used in the simulations are summarized in Table 1.

## Results

Before describing the simulation results, we provide the following definitions. A severed axon is considered to have successfully regenerated when it has regrown along the scaffold surface from the on-ramp to the off-ramp within 2 weeks of treatment. The success rate of axonal regeneration is the number ratio of successfully regenerated axons to all severed axons in the injury event (calculated by the current mathematical model). The growth rate of the regenerated axons is the average longitudinal extension velocity of all successfully regenerated axons (calculated as $\left(\sum_{k=1}^{N_{A}^{\prime }}V_{zk}\right)/N_{A}^{\prime }$, where *V*_*zk*_ is defined in Eq (6), and $N_{A}^{\prime }=N_{A}\times$ success rate). The growth rate should exceed 0.0025 μms^-1^ (1.5 mm per week) but should not exceed the physiological limit (~0.05 μms^-1^). The effective growth rate of the axons is the product of their success and growth rates, i.e., the longitudinal extension velocity averaged over all severed axons in the injury and repair process. It is given by $\left(\sum_{k=1}^{N_{A}}V_{zk}\right)/N_{A}$.

### Influence of the number of severed axons on regeneration

**Fig 3:**
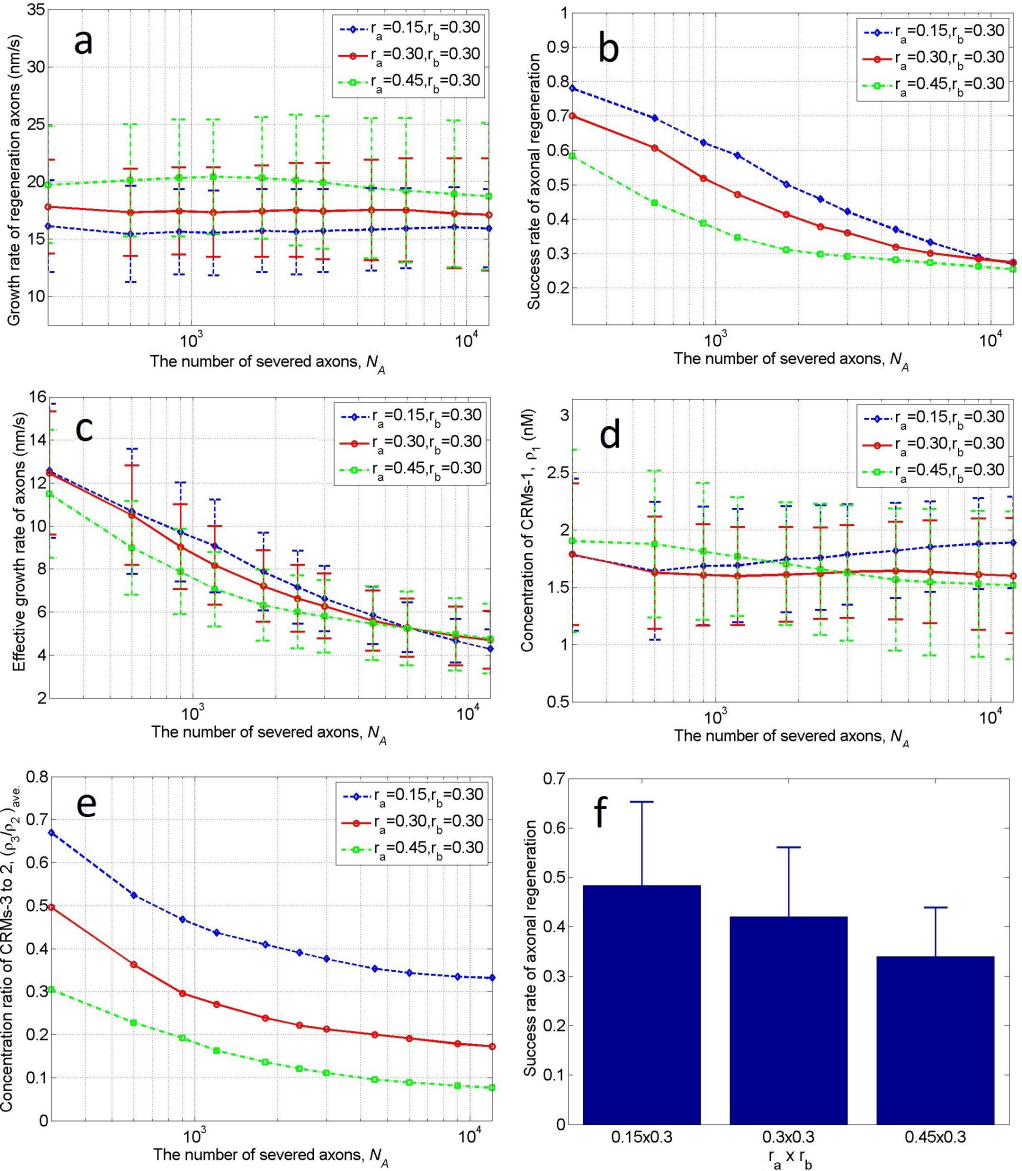
Influence of the number of severed axons on axonal regeneration. **a)** Growth rate of regenerating axons, **b)** success rate of axonal regeneration, **c)** effective growth rate, **d**) concentration of CRMs-1, and **e**) concentration ratio of CRMs-3 to CRMs-2, plotted as functions of log_10_ *N*_A_, where *N*_A_ is the number of severed axons. The curves in each panel were obtained on scaffold No. 1 (0.15 × 0.3), No. 2 (0.3 × 0.3), and No. 3 (0.45 × 0.3), respectively. **f)** Longitudinal coordinates in panel **b** averaged over the number of horizontal coordinates on scaffolds No. 1, No. 2, and No. 3 (left to right). Error bars indicate the standard deviations.

The number of severed axons *N*_A_ usually depends on the damage event. As previously mentioned, the maximum number of severed axons might be in the order of 10^−4^. In the following computer simulations, we varied *N*_A_ from 300 to 12000 and observed the consequent changes in the growth and success rates of post-SCI axonal regeneration. Next, we considered three scaffolds (No. 1, No. 2, and No. 3, sized at *r*_*a*_×*r*_*b*_ = 0.15×0.3, 0.3×0.3, and 0.45×0.3, respectively) and determined the most efficient size for axonal regeneration. For this purpose, we investigated how the slope of the on-ramp/off-ramp of the scaffold affects axonal regeneration. In this section, the density scale factors of the CRMs-1, CRMs-2, and CRMs-3 point sources on the scaffolds were considered as *η*_1_ = *η*_2_ = *η*_3_ = 1. In other words, the seed densities were constant and set to the same value (specifically, *γ*_1_ = *γ*_2_ = *γ*_3_ = 0.1 μm^−2^; see Table 1 and the subsection “Equivalent number of seeds on the scaffold and the point-source release rate” for details). All other parameter values are listed in Table 1. To clarify the scope and level of the model and the developed method, we first discuss the special case of *N*_A_ = 300. Fig 2 demonstrates the growth path and velocity of the regenerative axons and the CRM concentrations around the growth cone. By increasing *N*_A_ from 300 to 12000 at irregular intervals, we obtained a series of results similar to those demonstrated in Fig 2. These results are shown in Fig 3, which reveals how the number of severed axons influences axonal regeneration.

Fig 3A shows the effect of the number of severed axons *N*_A_ on the growth rate of the regenerating axons on each scaffold. Each point in the curves indicates the average growth rate of the surviving axons that grew along the scaffold from the on-ramp to the off-ramp. Axons that died or failed halfway were excluded. The error bars indicate the standard deviations of the averages. *N*_A_ does not significantly affect the growth rate provided that the axons can successfully regenerate. Moreover, the growth rate is proportional to the lateral size of the scaffold.

In Fig 3B, the success rate of axonal regeneration decreases with increasing *N*_A_. When *N*_A_ was moderately large (<9000), the slim scaffold achieved a high success rate. When *N*_A_ = 300, the success rate was approximately 35% higher on scaffold No. 1 than on scaffold No. 3; however, when *N*_A_ = 12000, the lateral size effect of the scaffold disappeared and the success rates on all three scaffolds declined to <30%. As the number of severed axons increased from 300 to 12000 on scaffold No. 1, the success rate was reduced by a factor of 3. From Fig 3A and 3B, we infer that a small on-ramp slope (i.e., a slim scaffold) increases the success rate of axonal regeneration up to a certain number of severed axons. When too many regenerative axons pass over the narrow bridge, the congestion lowers the growth rate. If success rate is more important than growth rate, the slim scaffold would be the first choice.

Fig 3C shows the effective growth rate of the axons (the product of the growth rate in Fig 3A and the success rate in Fig 3B as *N*_A_increases). Each point in the curves represents the growth rate of all regenerated axons, averaged over all severed axons, in the injury and repair process. The effective growth rate is a comprehensive index of axial sprouting and growth of axons after an SCI. Again, the slim scaffold is advantageous for axonal regeneration when *N*_A_≤6000.

In Fig 3D, the concentration of CRMs-1 sensed by the regenerating axons was relatively robust to both *N*_A_ and the lateral size *r*_*a*_ of the scaffold. However, the concentration ratio of CRMs-3 to CRMs-2 sensed by the regenerating axons heavily depended on both *N*_A_ and *r*_*a*_ (Fig 3E). Note that *N*_A_ and *r*_*a*_ are also highly correlated with the success rate of axonal regeneration (Fig 3B) and the effective axonal growth rate (Fig 3C). This suggests that the concentration ratio of CRMs-3 to CRMs-2 deserves more attention than it currently receives in SCI treatments.

Fig 3F averages the longitudinal coordinates in Fig 3B over the number of horizontal coordinates on scaffolds No. 1, No. 2, and No. 3. Again, the slim scaffold is beneficial for SCI treatments. In other words, irrespective of the number of severed axons, the slim scaffold is always the first choice for bridging. Therefore, the next two sections are devoted to scaffold No. 1 (slim scaffold with size 0.15×0.3).

### Influence of CRMs-1 point-source density on regeneration

**Fig 4:**
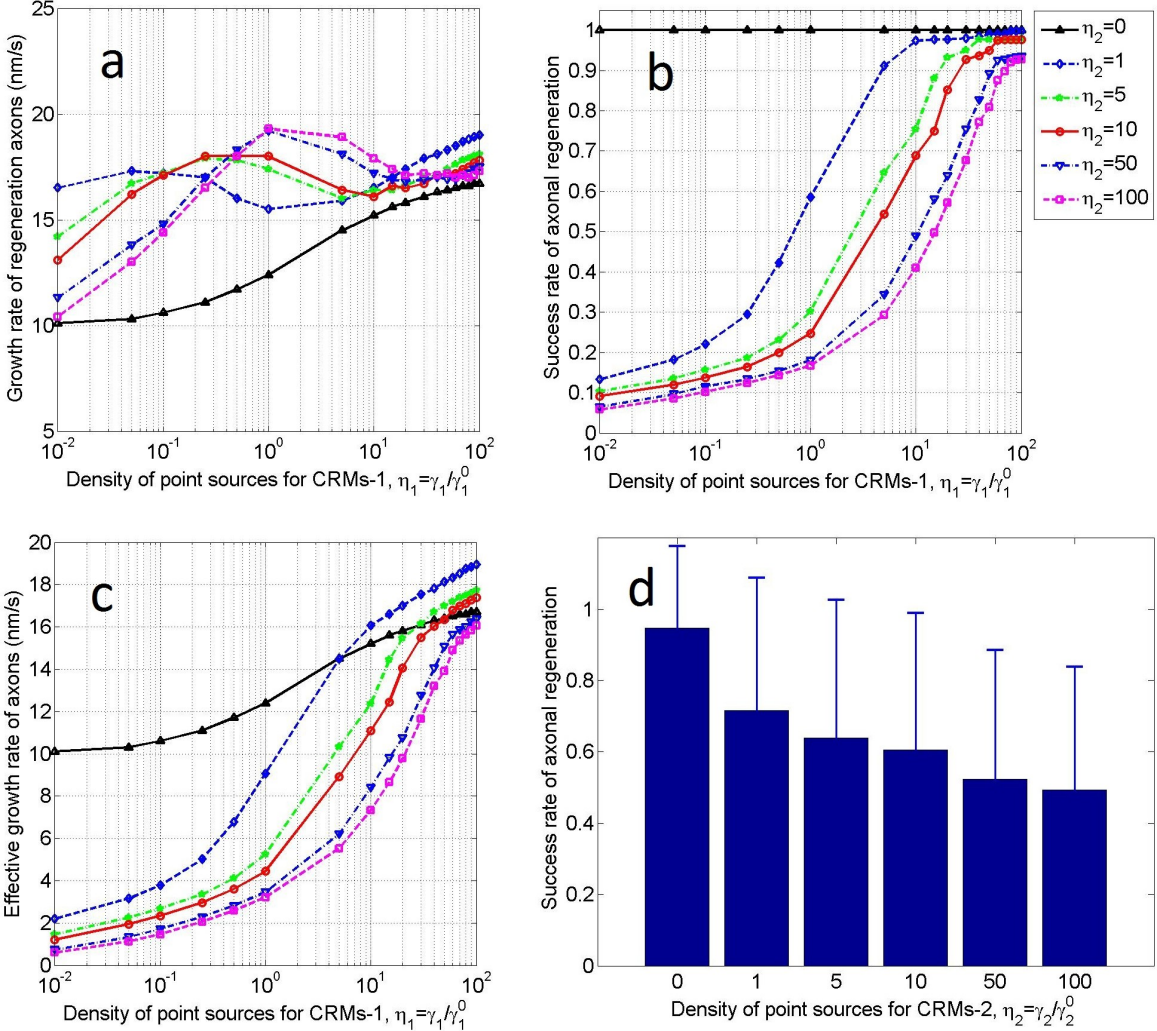
Influence of CRM1-1 point-source density on axonal regeneration. **a)** Growth rate of regenerated axons, **b)** success rate of axonal regeneration, and **c)** effective growth rate of axons as functions of the density (*η*_1_) of the CRMs-1 point source. Results are plotted for different densities (*η*_2_) of the CRMs-2 point source. The test scaffold is sized as *r*_*a*_×*r*_*b*_ = 0.15×0.3, the CRMs-3 point source density is *η*_3_ = 1, and the number of severed axons is *N*_A_ = 1200. **d)** Success rate of axonal regeneration averaged over *η*_1_ = 0−100 for each *η*_2_.

This analysis was performed on scaffold No. 1 (*r*_*a*_×*r*_*b*_ = 0.15×0.3). The prototype of the CRMs-1 point source is HA/LV-NT-3 and HA/LV-BDNF seeded on the off-ramp (Fig 1B,yellow) of the scaffold during fabrication (see subsection “Coating and seeds for the scaffold”). In each test, the seeding density of CRMs-1was $\gamma_{1}=\eta_{1}\gamma_{1}^{0}$ ($\gamma_{1}^{0}$ = 0.1 μm^−2^; see Table 1), where *η*_1_ was increased from 0 to 100 at irregular intervals. Alternatively, the CRMs-2 point source includes various inhibitory components, for example MAG induced by debris from the myelin sheath resulting from when axons were damaged, and CSPGs remaining on the scaffold surface, which cannot be easily controlled. Here, the density of the CRMs-2 point source was graduated through six levels: $\gamma_{2}=\eta_{2}\gamma_{2}^{0}$ ($\gamma_{2}^{0}$ = $\gamma_{1}^{0}$ = 0.1 μm^−2^, with *η*_2_ = 0, 1, 5, 10, 50, and 100). The density of the CRMs-3 point source was then expressed as $\gamma_{3}=\eta_{3}\gamma_{3}^{0}$. CRMs-3 represents the HA/ECM components (collagen I, fibronectin, and laminin I) coated on the whole surface of the scaffold during fabrication. Therefore, we set *η*_3_ = 1 (or $\gamma_{3}=\gamma_{3}^{0}$ = 0.1 μm^−2^, a constant). The number of severed axons can be selected from the feasible range (300–12000). Considering the available computer resources, we maintained *N*_A_ to be constant at 1200. Under these conditions, we performed the calculation and obtained Fig 4.

Fig 4A shows the growth rates of the regenerating axons as functions of density (*η*_1_) of the CRMs-1 point source for different densities (*η*_2_) of the CRMs-2 point source. Axonal growth is driven by the concentration gradient of CRMs-1 (∇*ρ*_1_) and is supported and inhibited by ∇*ρ*_3_ and ∇*ρ*_2_, respectively. Note that *ρ*_2_ and *ρ*_3_ are coupled to *ρ*_1_ through Eqs (8)–(10). Therefore, the growth rates of the regenerating axons are not simply proportional to *η*_1_ but change nonlinearly with increasing *η*_1_. Moreover, their extreme values depend on *η*_2_. Note that the growth rate was always lowest when *η*_2_ = 0, i.e., when no inhibitors were present in the injured microenvironment. This suggests that some remaining inhibitors (e.g., 0<*η*_2_≤1) are required to increase the growth rate. However, when *η*_1_>15 and *η*_2_>5, the growth rates were quite similar, suggesting that once the inhibitor level has exceeded a certain threshold, simply increasing the promoter levels is ineffective for increasing the growth rate.

Fig 4B shows the success rate of axon regeneration with increasing density (*η*_1_) of the CRMs-1 point source. First, when *η*_2_ = 0, i.e., when no inhibitor was present in the injured microenvironment, the success rate was 100% for all *η*_1_, except when *η*_1_ = 0 (i.e., when the success rate was 0). Next, for any fixed *η*_1_>0, increasing *η*_2_ decreased the success rate. Finally, when *η*_1_ was sufficiently high, the success rate of axonal regeneration exceeded 92%, regardless of *η*_2_. This suggests that reducing the inhibitor while increasing the promoter improves the success rate of axonal regeneration. The latter may be more important than the former because even if *η*_2_ reached 0, *η*_1_>0 would be required for axonal growth. However, as previously mentioned, a high success rate implies overcrowding of the growing axons on the scaffold, which eventually causes congestion and lowers the axonal growth rate. If the growth rate is excessively low, the axons might abort the growth process (in practice, growth cessation is due to unexpected causes) or an opportunistic time window for subsequent treatments may be missed. Therefore, when predicting axonal regeneration, the success rate must be balanced against the growth rate of the regenerating axons.

Fig 4C shows the effective growth rate of the axons. As previously mentioned, this index represents the sprouting and growth of the injured axons. Additionally, it is the product of the growth rates in Fig 4A and the success rates in Fig 4B. The effective growth rates were higher for *η*_1_>5 and *η*_2_ = 1 than for *η*_2_ = 0. If *η*_1_>50 could be achieved in practice, three treatments (*η*_1_>5and *η*_2_ = 1, *η*_1_>30 and *η*_2_ = 5, and *η*_1_>50 and *η*_2_ = 10) are preferred over the *η*_2_ = 0 treatment, and increasing *η*_1_ is much easier than achieving *η*_2_ = 0.

Fig 4D shows the average success rates of axonal regeneration over all *η*_1_ (0–100) for each *η*_2_. Regardless of *η*_1_, the success rates on scaffold No. 1 decreased with increasing *η*_2_ and the marginal effect was large for small *η*_2_. Again, this result highlights that reducing the inhibitors in the microenvironment is important for axonal regeneration. Note that when *η*_2_ = 0, the average success rate should be less than one (below 100%) because the success rate at *η*_1_ = 0 was zero. Thus, its logarithm (horizontal axis in Fig 4B) could not be defined. However, owing to non-zero *η*_1_ a unity success rate was achieved (Fig 4B).

### Influence of CRMs-3 on axonal regeneration

Finally, we numerically test the hypothesis that an over-eutrophic scaffold surface harms axonal regeneration.

**Fig 5:**
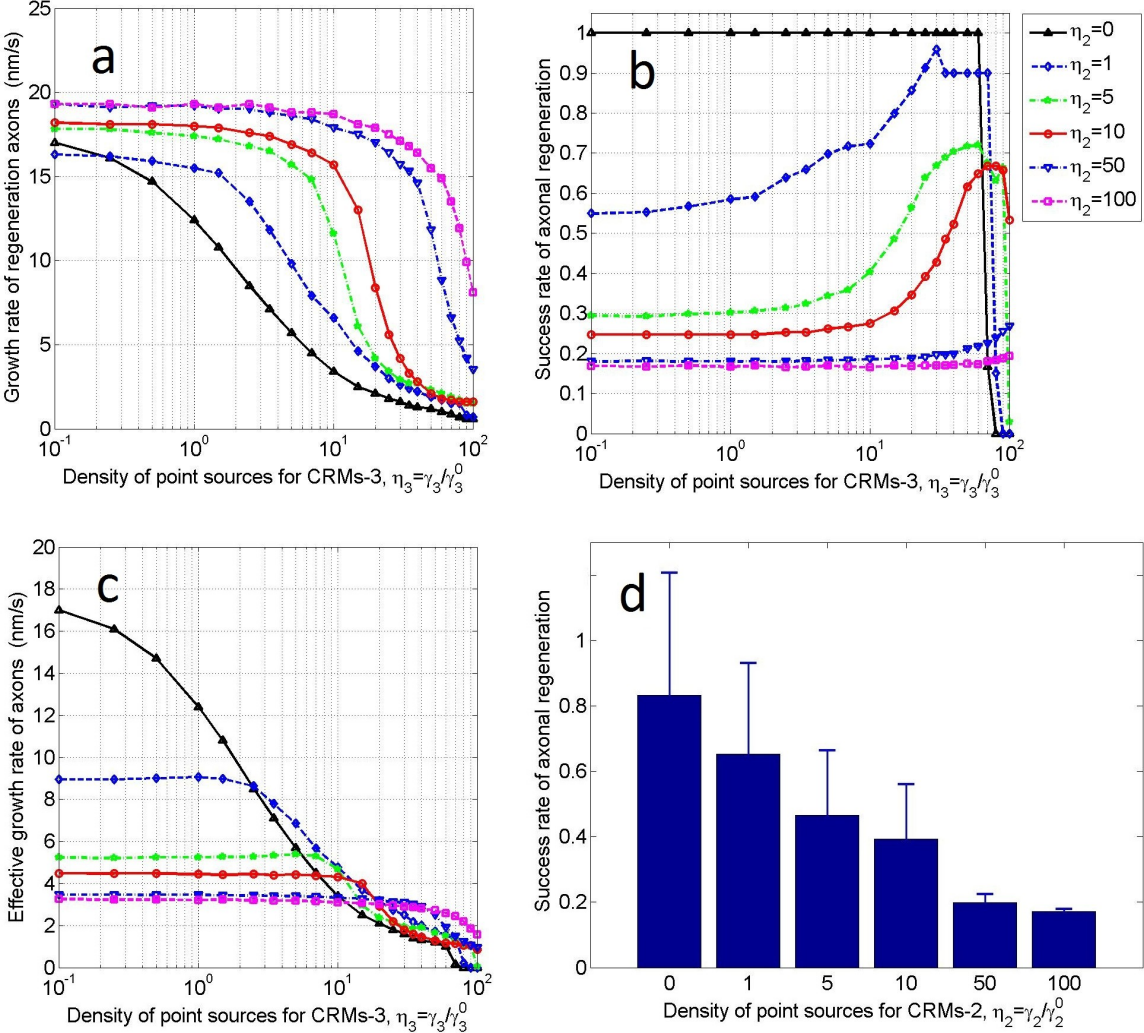
Influence of CRMs1-3 point-source density on axonal regeneration. **a)** Growth rate of regenerating axons, **b)** success rate of axonal regeneration, and **c)** effective growth rate of axons as functions of CRMs-3 point-source densityfor different densities (*η*_2_) of the CRMs-2 point source. The test scaffold is sized as *r*_*a*_×*r*_*b*_ = 0.15×0.3, the CRMs-1 point-source density is *η*_1_ = 1, and the number of severed axons is *N*_A_ = 1200. **d)** Success rate of axonal regeneration averaged over *η*_3_ = 0−100 for each *η*_2_.

The analysis was performed on scaffold No. 1 (*r*_*a*_×*r*_*b*_ = 0.15×0.3), and assumed a constant number of severed axons (*N*_A_ = 1200). The prototype of the CRMs-3 point source comprises HA/ECM components (collagen I, fibronectin, and laminin I) coated on the whole surface of the scaffold, as described previously. The coating density of CRMs-3, expressed as $\gamma_{3}=\eta_{3}\gamma_{3}^{0}$ with $\gamma_{3}^{0}$ = 0.1 μm^−2^(see Table 1), can be set during fabrication. In each test, density *η*_3_ was increased from 0 to 100 at irregular intervals. The CRMs-1 and CMRs-2 point-source densities were set as $\gamma_{1}=\eta_{1}\gamma_{1}^{0}$ (with $\gamma_{1}^{0}$ = 0.1 μm^−2^ and *η*_1_ = 1) and $\gamma_{2}=\eta_{2}\gamma_{2}^{0}$ (with $\gamma_{2}^{0}$ = 0.1 μm^−2^ and *η*_2_ = 0, 1, 5, 10, 50, and 100), respectively. The calculation results under these conditions are presented in Fig 5.

As shown in Fig 5A, the growth rates of the regenerating axons decreased with increasing *η*_3_ of the CRMs-3 point-source density, regardless of *η*_2_. Over a wide range of lower *η*_3_ values, the growth rates remained stable for all *η*_2_, except for *η*_2_ = 0. However, beyond a threshold *η*_3_ value, which increased with increasing *η*_2_, growth sharply declined. This supports the hypothesis that an over-eutrophic scaffold surface impedes axonal growth, and that certain inhibitors might naturalize the over-eutrophic effect and weaken or delay the harm.

Fig 5B shows the success rates of axonal regeneration varying as functions of *η*_3_ for each *η*_2_. When *η*_2_ = 0, i.e., when no inhibitor was present in the injured microenvironment, the success rate remained at 100% till the point when *η*_3_ = 60, and then dropped sharply to 16.67% at *η*_3_ = 70. Subsequently, the success rate fell to zero. At non-zero *η*_2_, the success rates first slowly ascended with increasing *η*_3_, then rose sharply before dropping to a low level, and finally reached zero. When *η*_2_ was high, the success rate curve began at a low level and ascended very slowly, forming a low plateau. For *η*_2_≥50, the success rate peaked only when *η*_3_ exceeded 100. This suggests that the use of an over-eutrophic scaffold surface to improve the success rate of axonal regeneration is inefficient and risks destabilization of the regeneration process.

Fig 5C plots the effective growth rate of the axons as functions of *η*_3_ for each *η*_2_. Applying an over-eutrophic scaffold surface to promote the sprouting and growth of axons after an SCI might be inefficient or even counterproductive.

Fig 5D shows the average success rates of axonal regeneration over *η*_3_ = 0−100 for each *η*_2_. Regardless of *η*_3_, the success rates decreased with increasing *η*_2_ on identical scaffolds and the marginal effect was greater at low *η*_2_ than at high *η*_2_. As discussed earlier, this result highlights the fact that reducing inhibitors in the microenvironment is important for axonal regeneration.

## Discussion

This study specifically aimed to create a microenvironment for axonal regrowth after SCI by mathematically changing the location of seeding on the scaffold, the density of the cells/factors seeded, and the size and shape of the scaffold. Therefore, we assumed a solid, spherical, multifunctional, biomaterial scaffold that bridges the rostral and caudal stumps of a completely transected spinal cord in a rat model for the calculations.

We assumed the body of the scaffold was made of PLG, the whole surface was coated with HA/ECMs and HA/LV-ChABC, and its off-ramp at the caudal area was additionally seeded with HA/LV-NT-3 and HA/LV-BDNF. These factors perform several functions. First, the on-ramp slope at the rostral area of the scaffold steers the growth cones of the regenerative axons onto the scaffold smoothly. Second, the factors seeded on the scaffold surface can be localized and sustained over a reasonably long period of time. The HA/ECM components adhere to the growth cones on the scaffold, supporting regenerative axons. HA/LV-ChABC secretes ChABC, which degrades CSPGs, inhibitory components from the glial scarsurrounding the injured tissue. The NT-3 and BDNF molecules released from HA/LV-NT-3 and HA/LV-BDNF at the off-ramp area can diffuse to the on-ramp area; this gradient can then guide the axonsat the rostral stump to grow along the outer surface of the scaffold and enter the caudal stump area, while also preventing the axons regenerating at the caudal stump from growing toward the rostral stump until they connect with axons emerging from the on-ramp area. That is, the scaffold forms a one-way bridge from the rostral to the caudal side, with a gentle slope of entry. From a mathematical point of view, the profile curve of the scaffold results in a small and continuous tangent slope, and the resulting concentration of all factors (promoters and inhibitors) on and around the scaffold, and varying along the span of the scaffold from one side to another, causes a monotonic increase. Provided that the scaffold is implanted upside down, the gradient of the resulting concentration or the direction of growth of axons will be reversed correspondingly, inferring from previous observations [18] that axons grow toward a site where additional cells have been injected, so either side of the bridge could be used as an injection site.Note that no matter which side of the bridge is used as the entry point, both motor and sensory neurons axons can cross the bridge because axons of both motor and sensory origins have previously been found within a tunneled scaffold [14].

Several scaffolds for SCI have previously been described [3,4,5,6,7], such as cell grafts [8,9], tubes or conduits [10], and cylinders with tunnels/linear pores or filled with fibers, either randomly or in alignment. Of these, cell grafts comprise natural soft tissues, with conduits that are empty or contain soft matrices and/or cells, and with a wide diameter, making it easy for regenerative axons to enter them, with no need to consider an entry slope. However, axons which entered these grafts were often trapped in them and rarely re-entered the host tissue. It was commonly believed that fine physical guidance was required within the cell grafts and conduits. Therefore, cylinders with tracks (a rolled-up nanofiber sheet or film) [10,11] or tunnels (via modeling) [14,19], combined with seeding of cells/factors [12,13,14,15,16,18,19], emerged and mostly replaced the use of cell grafts and conduits. However, not only did regenerative axons still get trapped in the guideways [10,11,12,13,14,15,16], but congestion was also observed at the entries [19]. This trapping, according to the current study, is caused by the over-eutrophication in and/or on the scaffold, and the lack of additional chemotactic factors close to either side of the scaffold. Even if these factors are uniformly distributed throughout whole scaffold during fabrication, because the factors diffuse easily from the two ends of the scaffold, they reach the middle of the scaffold after a period of time, which attracts regenerating axons from both ends to the middle. Therefore, the scaffold becomes a two-way channel, leading the axons to grow toward each other, probably resulting in their intersection. However, there is little direct evidence to show that such intersected axons can form synaptic connections. In this situation,the factors on the scaffold are more likely to be a barrier against the regenerating axons. Fortunately, the axons can sometimes break through this barrier [19], as was simulated in this study, and re-enter the host tissue. This is typically due to additional chemotactic factors being set close to either the caudal [19] or rostral stumps [18]. Alternatively, factors (known or unknown) contained in and/or on the scaffold might form (intentionally or unintentionally) a consistent gradient along the span of the bridge; there may also be a small probability that some unknown factors from host tissues form a beneficial gradient for axonal regeneration. Based on the typical situation [19], we inferred that, according to the principle of chemotaxis of axons, only if the peak of the barrier is much lower than the summit formed by the chemotactic factors at either end, the axons can break through the barrier.

However, another barrier that exists for scaffolds with tracks or tunnels is the entry obstruction that lowers the number of axons entering into the spaces or pores of the scaffoldbecause of the absence of a uniform entry slope. For tunneled scaffolds [14, 19], the slope of entry is small or zero only for those axons whose growth cones face to the pores, where the axonal incidence angle *α* = 0 (i.e., the slope = tan(0) = 0); whereas for the other axons at the entry, the slope is abrupt or infinite, due to *α* = ±*π*/2 (i.e., slope = tan(±*π*/2) = ±∞). This prevents the axons from regenerating straight ahead and reduces the number of axons entering the tunnels. Pore size is another parameter that affects the ability of axons to pass through a scaffold, and while investigating optimal pore size is difficult, the present model can be modified to study this. Once the number of axons entering a scaffold is small, the number of axons exiting will be much lower, according to previous observations [19]. The present spherical scaffold described by us is not only simple but also, at least theoretically, lacks the shortcomings inherent in other porous scaffolds.

The proposed scaffold could be used to replace previous scaffolds used in rat models for SCI repair [10,11,16], to bridgea gap of approximately 3 mm. It is worth noting that the present scaffold might offer additional possibilities. The complexesseeded on the caudal area that express NT-3 and BDNF, as well as being diffusible chemoattractants for attracting axonal growth cones, have enhanced oligodendrocyte survival and axon myelination [24,51,52,53]. NT-3 and BDNF may also enhance a number of other processes. For instance, BDNF has been associated with a reduction in the inflammatory response, including a reduction in astrocyte numbers [54], which further aids axon regeneration. Whilefabrication of our scaffold might be difficult, and the use of hydroxylapatite (HA) is probably not the best choice due to the risk ofMilwaukee shoulder [34], we do provide a prospective direction for SCI repair.

Our mathematical model embodies the geometry, chemistry, and physics of the system under investigation. The shape and size of the scaffold provide the geometrical boundary conditions that constrain the growth cone’s movement or the regenerative axon growth. While the inclusion of all chemical factors in a single model is difficult, a coarse-grained method can be used to obtain a balance between the reduction in the number of factors and the retention of the chemical properties. That is, the CRMs on and around the scaffold (assuming it has been implanted) were classified into three types with different chemical properties: the CRMs-1 group comprised chemoattractants of axonal growth (NT-3/BDNF secreted by seeded HA/LV-NT-3 and HA/LV-BDNF at the off-ramp area); the CRMs-2 group comprised chemorepellents of axonal growth (compounds produced by the injured tissue, such as Nogo-60, MAG, and OMG, and the remnant CSPGs that are not neutralized by ChABC released from seeded HA/LV-ChABC); and the CRMs-3 group comprised molecules released from the coated HA/ECM components, which support axonal growth. Among these groups, CRMs-1 plays the leading role in axonal regeneration, whereas CRMs-2 and CRMs-3 provide a balanced and coordinated effect, and interact with CRMs-1 (for which, CRMs-2/3 was formulated as a function of CRMs-1). The physical and biophysical aspects of the model are the Fickian diffusions and reactions of the CRMs and the chemotaxis of the axonal growth cone motility. Under CRMs-1 and CRMs-3 gradients, the regenerative axons elongate toward the target cells, whereas under the CRMs-2 gradient, they retract.A similar chemotaxis pattern has been validated via experiments [35,36,37], and mathematicallymodeledfor studying axonal growth in neural development [28,29,30,31]. Note that axonal growth/regrowth follows the same processes observed experimentally both in development and injury, in vivo and in vitro, because in both cases the experiments always initially cause damage to the nerve cells, for instance during surgery or separation. The difference between them, however, is in the degree of damage, and in the age of the experimental subjects. Therefore, a theoretical model for axonal growth during development can be modified for studying SCI. In fact, the present model is mathematically similar to those described in the literature [28,29,30,31].

By applying our mathematical model to the theorized scaffold, we numerically studied the influence of the number of severed axons; the slope of the on-ramp of the scaffold (which is related to the lateral size of the scaffold); and the concentrations and gradients of the CRMs (which are related by their seeding densities to the survival and growth of the regenerative axons).

Axonal regeneration was evaluated based on the growth and success rates of the regenerated axons. A severed axon has successfully regenerated when it has regrown along the scaffold surface from the on-ramp to the off-ramp within 2 weeks of the treatment. The success rate defines the number ratio of the successfully regenerated axons to all axons severed in an injury event. The growth rate is the average longitudinal extension velocity of the successfully regenerated axons.

It should be noted, however, that our model is highly idealized, for example, the basis data we used for the calculation of growth cone velocity were not derived from trauma tissue, which might be spatially different in physics and chemistry, and not be reflected by CRMs-2. The intersections and/or tangles between regenerating axons and the energy expenditure for axonal growth were not modeled in the current study. The cut to generate a spinal cord injury should not be too wide (>1 cm) for using this model to predict SCI because the effective diffusion distance of CRMs-1 might be limited to approximately 1 cm by Fick’s first law, on which this model is based. Only an in vivo verification will show whether these equations will hold.

## Conclusions

A solid, spherical, multifunctional, biomaterial scaffold is assumed to bridge the rostral and caudal stumps of the spinal cord in a completely transected rat model, thereby promoting the entry of regenerative axons from the rostral stump into the caudal stump tissue at the opposite side of the scaffold.

Three scaffold shapes (slim, round, and stocky) were investigated in our simulations. Among them, the slim shape benefited axonal regeneration the most by presenting a small slope at the on-ramp area. However, if the success rate becomes too high, numerous regenerative axons crowd into a narrow area, causing congestion and resulting in a reduced growth rate. The stocky scaffold induced the opposite effect, and the round scaffold induced intermediate effects. When success rate is more important than growth rate, the slim scaffold should be the first choice.

The number of severed axons in an injury event (between 300 and 12000) does not significantly affect the growth rate of the regenerated axons, but does influence the success rate of axonal regeneration (particularly, the success rate decreases with increasing number of severed axons).

Among the three types of chemical treatments, raising the CRMs-1 (NT-3 and BDNF) level while reducing the CRMs-2 level (CSPGs and other chemorepellents) benefited the success and growth rates of axonal regeneration the most. Physically, the CRMs-1 level was increased by increasing the seeding density of HA/LV-NT-3 and HA/LV-BDNF on the off-ramp of the scaffold, whereas the CRMs-2 level was reduced by increasing the seeding density of HA/LV-ChABC over the entire scaffold surface. However, raising the CRMs-3 (ECM components) level by increasing the density of HA/ECM components over the entire scaffold surface may create an over-eutrophic surface that harms axonal regeneration.

The theoretical predictions made in this study need to be experimentally validated in the future. In principle, the current tool can be easily modified for predictions regarding scaffolds with other architectures.
